# Identification of somatic mutations in cancer through Bayesian-based analysis of sequenced genome pairs

**DOI:** 10.1186/1471-2164-14-302

**Published:** 2013-05-04

**Authors:** Alexis Christoforides, John D Carpten, Glen J Weiss, Michael J Demeure, Daniel D Von Hoff, David W Craig

**Affiliations:** 1 Translational Genomics Research Institute, Neurogenomics Division, Phoenix, AZ 85004, USA; 2 Department of Biomedical Informatics, Arizona State University, Tempe, AZ 85284, USA; 3 Translational Genomics Research Institute, Integrated Cancer Genomics Division, Phoenix, AZ 85004, USA; 4 Virginia G Piper Cancer Center, Scottsdale, AZ 85258, USA; 5 Clinical Translational Research Division, Translational Genomics Research Institute, Scottsdale, AZ 85259, USA

**Keywords:** Cancer genomics, Next generation sequencing, Somatic mutation detection

## Abstract

**Background:**

The field of cancer genomics has rapidly adopted next-generation sequencing (NGS) in order to study and characterize malignant tumors with unprecedented resolution. In particular for cancer, one is often trying to identify somatic mutations – changes specific to a tumor and not within an individual’s germline. However, false positive and false negative detections often result from lack of sufficient variant evidence, contamination of the biopsy by stromal tissue, sequencing errors, and the erroneous classification of germline variation as tumor-specific.

**Results:**

We have developed a generalized Bayesian analysis framework for matched tumor/normal samples with the purpose of identifying tumor-specific alterations such as single nucleotide mutations, small insertions/deletions, and structural variation. We describe our methodology, and discuss its application to other types of paired-tissue analysis such as the detection of loss of heterozygosity as well as allelic imbalance. We also demonstrate the high level of sensitivity and specificity in discovering simulated somatic mutations, for various combinations of a) genomic coverage and b) emulated heterogeneity.

**Conclusion:**

We present a Java-based implementation of our methods named Seurat, which is made available for free academic use. We have demonstrated and reported on the discovery of different types of somatic change by applying Seurat to an experimentally-derived cancer dataset using our methods; and have discussed considerations and practices regarding the accurate detection of somatic events in cancer genomes. Seurat is available at https://sites.google.com/site/seuratsomatic.

## Background

The rise of next-generation sequencing (NGS) brought with it a demand for robust tools for variant detection from sequencing read data, typically after the data has been aligned against a reference sequence. A variety of mature analysis tools, workflows and approaches are already available to the scientific community, and the detection of common types of genomic variation in haploid and diploid genomes is a rapidly maturing area of development [1-3].

More recently, NGS has been employed in order to provide new insight into the genetic mechanisms of cancer, as the technology enables the exploration of tumor genomes in previously infeasible levels of detail. Among many examples, researchers have used it to examine the patterns of genomic alteration in non-small-cell carcinoma [4] and melanoma cell lines [5], to discover novel and possibly tumorigenic mutations in the acute myeloid leukemia genome [6], and have even used findings to inform clinical treatment of a patient with acute promyelocytic leukemia [7].

Cancer cells have deviated from the normal (germline) genome of the organism by acquiring and selecting for a set of mutations which enable them to grow rapidly and invasively, to resist regulation and/or possibly to metastasize [8]. These changes can be simple single-base mutations to more complex genomic gain, loss or structural change events. The changes can then trigger the cancer process by modifying the function of a protein (e.g. disabling a tumor suppressor gene, or activating an oncogene), silencing a gene’s transcription or affecting a gene’s transcriptional affinity. In order to separate germline variants from these acquired (somatic) mutations of the malignant tissue, many studies have elected to sample and sequence both the tumor tissue and separate tissue with a normal genomic profile from the same individual. The tumor-unique variants are then identified; for this process, researchers have often decided to use established standard variant detection tools on both sequenced genomes, and then apply heuristic filtering methods to establish a set of confident calls out of the two result sets [5,6].

Cancer genomes, however, pose unique challenges to variant detection from NGS data that define the effectiveness of standard methods. Aneuploidy, massive genomic amplifications and structural variations are common in cancer [9]; consequently, the assumption of a diploid genotype (made by most variant calling software) is no longer sound. This is further complicated by the fact that specific variations are often rare or unique to each cancer, and cannot be compared to a ‘golden standard’ genomic profile, even within the same cancer type. Some cancers are heterogeneous, with some somatic variants appearing only in small cell subpopulations of the malignant tissue. Subpopulation variants however may be critical to tumor viability [10] and are therefore interesting to researchers. Finally, tumor biopsies often suffer from degradation and contamination with non-malignant tissue to varying degrees, depending on the type of the tumor and the biopsy method [9]. Generally, it becomes very likely that analysis and downstream research would be hindered by a high false-negative rate by variant calling algorithms that do not take these properties of tumor physiology into consideration.

Presently, tools have been developed or extended with cancer genomics specifically in mind. OncoSNP [11] utilizes a specialized Bayesian framework for detection of genomic aberrations in cancer, but is designed for the analysis of single nucleotide polymorphism (SNP) microarray data. SNVMix [12] is one of the first efforts that serves NGS studies, and attempts to resolve point mutations in aneuploid genomes using a binomial-mixture model that is optimized using expectation-maximization. SNVMix does not currently support paired normal/tumor analysis, however. Other approaches include somatic small variant tool Strelka [13], the new somatic extensions in the variant-detection tool VarScan [14], and the specialized Bayesian tool SomaticSniper [15]. All of the methods mentioned focus on small genomic events, and none provide specific support for integrated genome/transcriptome analysis, structural variation detection or detection of allelic imbalance.

We present a generalized Bayesian-based approach for detecting genomic aberrations unique to one sample set with the goal of extending beyond detection of point mutations. Our methods are founded on Bayesian statistical theory and extract a probability value for a somatic event by comparing the likelihood of the available evidence against all possible explanations (models), and adjusting the likelihoods with a prior-knowledge probability for each explanation. While we compare the normal genome against models with certain assumptions such as diploidy, the assessment of the tumor data is only in reference to its similarity with normal data. Increased evidence in either the normal or tumor profile will therefore increase sensitivity by either providing more evidence towards a somatic change, or more evidence for lack of variation in the normal. Since this model does not assume a particular distribution of variant evidence in the tumor, it is robust to changes that appear in low allelic frequencies, as would possibly be the case with aneuploid genomes or sequenced samples that were contaminated by stromal cells. Similarly, the detection of allelic imbalance is performed by comparing the likelihood of a ‘balanced’ transcription and the expected evidence presentation on heterozygous loci, against the possibility of the tumor/normal variant proportions being independent.

## Results

We developed a Bayesian-based analysis framework for identifying genetic mutations specific to one dataset, as is the case of somatic mutations within tumors for tumor/normal pairs. The framework (which we call *Seurat*) considers the joint probability that a variant is existent within the tumor dataset but not within the normal dataset.

Seurat iterates through each nucleotide in the reference sequence and examines any evidence from aligned reads at that locus. The evidence is then split in two classes: “Variant” and “Normal” (this process is detailed in the Methods section). The method can also be applied with evidence from a sliding window over a reference sequence, or evidence from discrete annotated regions such as exons or whole genes. Depending on the somatic change that we are attempting to detect, a unit of “Variant” evidence can be defined as an aligned base that does not match the reference (indicating a base substitution), a gap in the alignment (indicating an insertion or deletion), a mate-paired read with an atypical mate alignment distance (indicating larger structural variance), or a read that aligns with unexpected orientation (indicating an inversion). The normal genome is then tested for normality given prior expectations of variant evidence occurring due to error. Then, assuming lack of genomic events on the normal genome, we proceed to test the tumor for a proportion of variant evidence that significantly differs from the proportion in the normal genome. Such dissimilarity would then signify a somatic event.

### Implementation

The methodology could conceivably be implemented on top of a wide variety of sequence “walkers” that iterate through aligned short sequence fragments. Our implementation is a module for the Genome Analysis Toolkit (GATK) framework [3]. The functionality is exposed through a command line interface that requires as input a reference sequence file in the FASTA format, a reference-ordered data (ROD) file containing gene annotations, and two Binary Alignment/Map files (BAMs) with the data for the normal and tumor genomes. Output is generated in two text files: One is a list of focal somatic variants presented the commonly-used Variant Call Format [16]; the other is a separate catalog of larger detected events.

The implementation design allows for the creation of Seurat “sub-modules” that can utilize the core methods presented for the detection of other small, gene-wide or exon-wide events that may be supported in the future. The currently available feature detection modules are listed in Table 1. Seurat is open-source software, and is available with a free license for academic and non-commercial use at https://sites.google.com/site/seuratsomatic.

**Table 1:** Biological features currently supported by the Seurat software, and their respective input files

**Feature**	**Input data sources**	**Optional data sources**
**Somatic base substitutions**	1. Normal DNA BAM	Normal RNA BAM
	2. Tumor DNA BAM	Tumor RNA BAM
**Somatic insertions/deletions**	1. Normal DNA BAM	Normal RNA BAM
	2. Tumor DNA BAM	Tumor RNA BAM
**Somatic loss of heterozygosity**	1. Normal DNA BAM	Normal RNA BAM
	2. Tumor DNA BAM	Tumor RNA BAM
**Allelic imbalance**	1. RNA BAM	--
	2. DNA BAM	
**Somatic structural variance**	1. Normal DNA BAM	--
	2. Tumor DNA BAM	

### Evaluation of somatic mutation detection accuracy using simulated data

Point mutations are aberrations that are frequently observed in cancer genomes, and have been long studied and causally linked with driving carcinogenesis or tumor progression, typically by causing the activation of an oncogene [5,17]. The substitution of a single base within the coding region of a gene may result in an amino-acid change or premature truncation of a protein, and mutations in other regions can cause splicing errors, transcription silencing, or other potentially adverse effects that can trigger abnormal cell proliferation. Aside from base substitutions, small genomic insertions and deletions (less than 100 bp) are also common and can disable or alter the result of gene transcription. The effect can range from the addition or removal of amino-acids to the translated protein sequence to the creation of frameshift event, where the interpretation of codons during translation is changed completely downstream of the variation.

Seurat detects point mutations by using the counts of aligned bases that support a variant genotype (e.g. A non-reference nucleotide or insertion/deletion evidence), versus the total number of aligned bases. Base substitutions are generally the easiest genomic alterations to detect in alignment data. However, systematic errors are still often introduced by the alignment process, particularly in homologous regions. Two very useful metrics that are generally provided by contemporary aligner software are the mapping quality and base quality scores. Mapping quality refers to the confidence that the aligner software package assigns to its own alignment call, while base quality scores refer to the sequencing instrument’s confidence in assigning a genotype to each sequenced nucleotide. Seurat by default filters data with a mapping or base quality score that is lower than 10 in the Phred scale (corresponding to <90% confidence of a correct call). Another common issue is strand bias, where the only evidence supporting the variant are reads aligning in just one direction. As this usually indicates a mapping artifact, we have added an optional filter which requires each reported candidate variant to be supported by at least one read in each direction in order to reduce our false positive frequency. We also support filtering based on per-Base Alignment Quality, which is a post-alignment calculated metric for the probability of a base mismatch being the result of a misalignment [18].

Typically, failure to detect genetic variants in NGS data is a result of the inability of the alignment software to map the sequenced variant reads to the genomic region, or a failure to sample the variant allele sufficiently or at all [1]. This is further complicated in cancer genomes, where the somatic mutations may be present in only a subset of the biopsied genetic material [9]. Furthermore, we must attempt to identify variants that are only in one of the two genomes, thus a somatic mutation satisfies that (1) it is not the germline dataset and (2) it is present in the tumor set. Finally, we do not get to presume diploidy or lack of normal-tissue contamination in cancer, so variant evidence does not necessarily appear in the often-expected frequencies of 0%, 50% or 100%.

False-positive somatic mutation calls from paired genomes are also a non-trivial concern and may derive from multiple sources. First, instrument and alignment errors can occasionally present themselves as consistent and sufficient evidence for variance. Second, it is possible that a germline variant (i.e. a SNP) can fail to be detected in the normal genome, and at the same time be successfully detected in the tumor genome by the analysis software. This variant will then be misrepresented as a tumor-specific mutation [9].

Both false-positives and false negatives from the above example sources of error can theoretically be addressed through sufficiently high genomic coverage, coupled with methods that can robustly leverage the additional data. We have developed a somatic change detection framework that addresses these considerations. Coverage at any given place in the genome is variable however, and low coverage regions may still lend to false-positives when coverage is not high enough to identify germline variants.

To evaluate Seurat’s somatic mutation sensitivity and specificity under a range of realistic conditions, we created a simulated cancer dataset using aligned genomic sequence data from the 1000 Genomes Project [19]. We appropriated a set of known mismatching polymorphisms between two unrelated genomes to be an emulation of known somatic point base substitutions. We also used two lists of known true negatives, one for each source of false positive calls described above (reference genotype in both samples/ variant genotype in both samples).

#### Effect of normal and tumor coverage on detection performance

**Figure 1:**
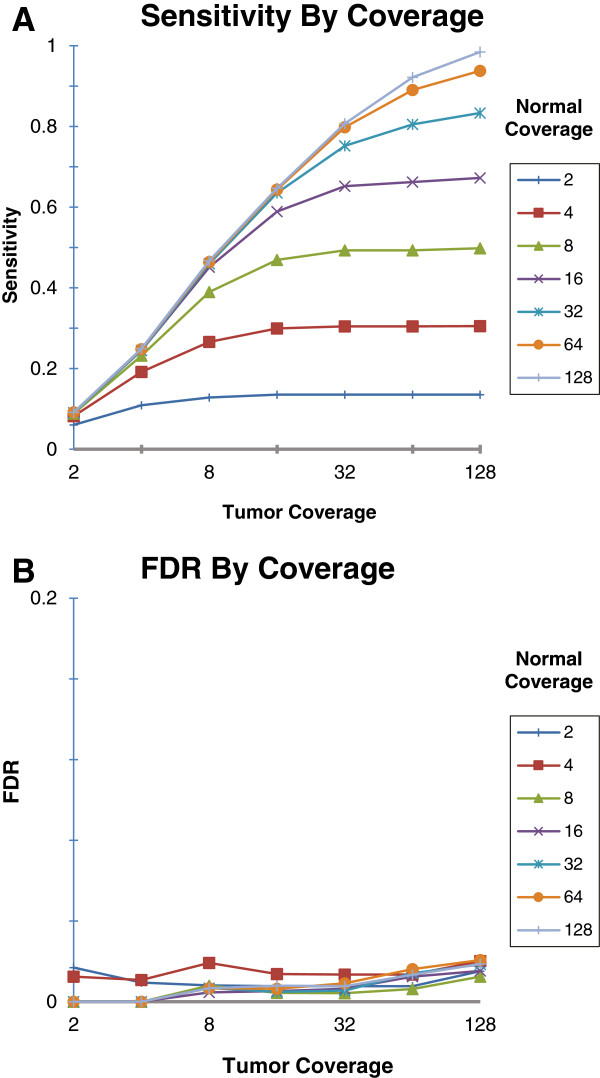
**Performance of Seurat’s somatic point mutation detection with varying genomic coverage.** Legend: The sensitivity **(A)** and false discovery rate **(B)** for Seurat’s somatic point mutation detection method, evaluated on simulated cancer genome data with no simulated normal tissue contamination. Each series represents the coverage used for the ‘normal’ genome data set, and the x-axis represents the ‘tumor’ genome average coverage.

The simulated data for both normal and tumor were down-sampled to generate sets with varying average coverage, in order to explore the effect of sequencing throughput to accuracy. Seurat was then used to analyze each combination of down-sampled normal/tumor pairs, and the output for each combination was compared to the ground truth sets to measure sensitivity and false discovery rate (FDR), as we present in Figure 1. We have found that increases in normal and tumor sequencing output both translate to a rise in mutation sensitivity, and the best improvement gradient comes from the simultaneous raise in both normal and tumor coverage, peaking at ~0.98 at the 128× level, while maintaining a FDR under 0.02. This ability to increase both sensitivity and specificity by sequencing more of just the normal genome can be very useful in cases where genetic material from the tumor is scarce or hard to acquire.

#### Effect of heterogeneity on detection performance

From the above simulated normal/tumor dataset, we derived another series of datasets where the tumor sequence was now admixed with normal sequence throughout the spectrum of possible ratios, while total sequence remained constant at 128×. Along with Seurat, we used this range to evaluate three additional popular and publically-available normal/tumor analysis packages: Strelka [13], SomaticSniper [15] and Varscan 2 [14]. Details and paremeters for this comparison can be found in [Additional file 1].

**Figure 2:**
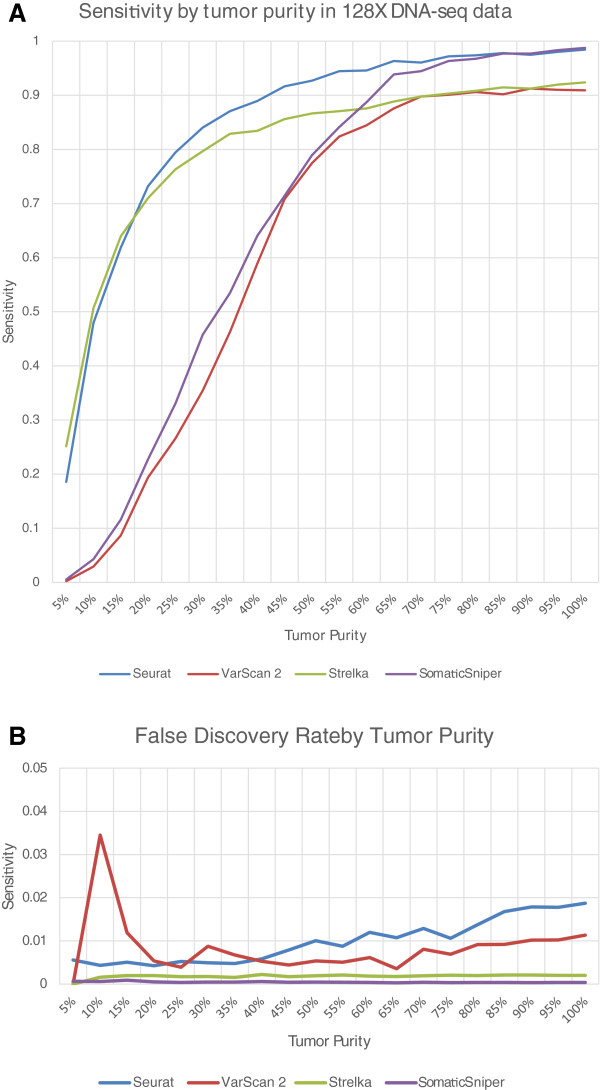
**Performance of somatic point mutation detection with varying tumor purity.** Legend: The sensitivity **(A)** and false discovery rate **(B)** for Seurat, VarScan 2, Strelka and Somaticsniper, given tumor DNA data of varying simulated tumor purity. Seurat reaches 90% sensitivity at ~45% tumor purity in sequence data with average genomic coverage of 128 × .

Figure 2 shows our results. We found that Seurat is able to discover >90% of the simulated mutations for tumor purity as low as ~45%. Both Seurat and Strelka are able to call ~50% of variants at 10% purity, while the sensitivities of SomaticSniper and VarScan are significantly lower at the lower purity levels with these specific datasets and scenarios. It’s important to highlight that any comparison is subject to biases of the individual tests and do not imply that a software is ‘better’ or ‘worse’ for a specific situation. However, these comparisons do shed light on strengths and weaknesses of Seurat within the confines of tumor heterogeneity.

### Application to experimental cancer sequencing data

We applied Seurat to a cancer sequencing dataset from a patient diagnosed with a rare, late-stage lymphoma. Sequencing was performed on an Illumina Genome Analyzer IIx instrument, sequence alignment was performed using the CASAVA software package and the resulting BAM files were processed by our implementation. Analysis with Seurat was performed using 24 Intel Xeon Harpertown cores at 2.83 GHz (4 GB of RAM allocated to each core) and a Lustre distributed file system for high-performance input/output. The process was completed in approximately 5 hours, and Seurat produced locus and probability output for candidate somatic base substitutions, indels, loss of heterozygosity and structural variation events. We summarize our results and their additional evaluation in Table 2. The high transition/transversion ratio, low non-synonymous/synonymous ratio and low dbSNP rate indicate Seurat’s high specificity and low false discovery rate, and variants with a Phred-scale quality of over 20 predictably perform even better in these metrics.

**Table 2:** Summary of analysis results from the application of Seurat on an experimentally derived cancer dataset

**Metrics(example tumor/normal dataset)**	
Average genomic coverage on normal tissue genome	55×
Average genomic coverage on tumor tissue genome	40×
Somatic base substitutions	29526
Somatic base substitutions (Quality > 20)	17044
Transition/Transversion ratio for somatic base substitutions	1.433
Transition/Transversion ratio for somatic base substitutions (Quality > 20)	1.922
dbSNP build 135 rate	0.146
dbSNP build 135 rate (Quality > 20)	0.088
Somatic insertions	1430
Somatic deletions	4067
Somatic structural variance sites	272
Somatic loss of heterozygosity sites	1523
Non-synonymous/Synonymous mutation ratio	0.00435

Detailed Legend: Summary of somatic mutation analysis details from the application of Seurat on a normal/tumor genome pair of a patient with a rare lymphoma. The dbSNP rate refers to the proportion of candidate somatic variants that are included in the public genomic variation database dbSNP [20]. This number is an indicator of known germline variants that were falsely identified as tumor-specific. The calculation of the transition/transversion and the non-synonymous/synonymous variant ratios was performed using snpEff [21].

### Loss of heterozygosity (LOH)

Loss of heterozygosity (LOH) refers to a genomic deletion that removes a functional copy of an allele in a cell. In the context of cancer, LOH events are usually important in occasions where the second allele of a gene was already mutated or inactive; typically this gene would be a known tumor-suppressor gene that would now become completely disabled. This can be an early event or a necessary condition for the instigation of cancer, and LOH of the *TP53* and *RB* tumor-suppressor genes have been studied for their role in a wide variety of human cancers [22,23].

LOH events can be detected using a similar way as base substitutions, but the expectation of variant allele evidence is rather placed on the normal-tissue genome data, while the tumor genome is expected to be ‘variant-free’. We have observed that the proportions of reference to variant evidence can vary wildly between datasets, signifying that the relative alignment “affinity” of each of the alleles is highly sensitive to subtle changes in protocol (i.e. sample preparation, sequencing environment, revisions of the alignment software). Contrasting our somatic mutation method, we decided to not use the evidence from the normal genome to “update” the idea of the expected genotype – systematic shifts were introducing a very high rate of false positives.

### Structural variation

It is also possible to observe major structural genomic changes via alignment data. For mate-paired sequencing, the aligner software will attempt to match the two sequenced fragments within the insert size distance and orientation that is expected by the sample sequencing biochemistry and protocol. If that is not possible, the fragments will be aligned independently and the resulting alignment file will include the information about the unexpected event.

Under our method, each ‘abnormal’ fragment can count as a piece of variant evidence. Abnormal fragments that belong in the same variant ‘subclass’, (such as reads whose mates all align in the same trans- region) can for example be evidence of a genomic translocation. A significant number of abnormal reads with properly oriented mates in the same chromosome can be the result of a large deletion, while a cluster of abnormally-oriented reads can be because of an inversion event. Using our somatic mutation formula, a somatic structural event is once again indicated by such evidence appearing primarily and confidently in the tumor.

## Discussion

We have presented a paired genome analysis method and accompanying software package for cancer genomes and transcriptomes. The Bayesian approach and the use of beta-binomial probability distributions were shown to be useful in modeling the uniqueness of genotype discovery in cancer. Admixed genomes, as well as unpredictable ploidy in tumor DNA, can be accounted for; and higher coverage increases the method’s ability to discover somatic variants with very low allelic frequency.

**Figure 3:**
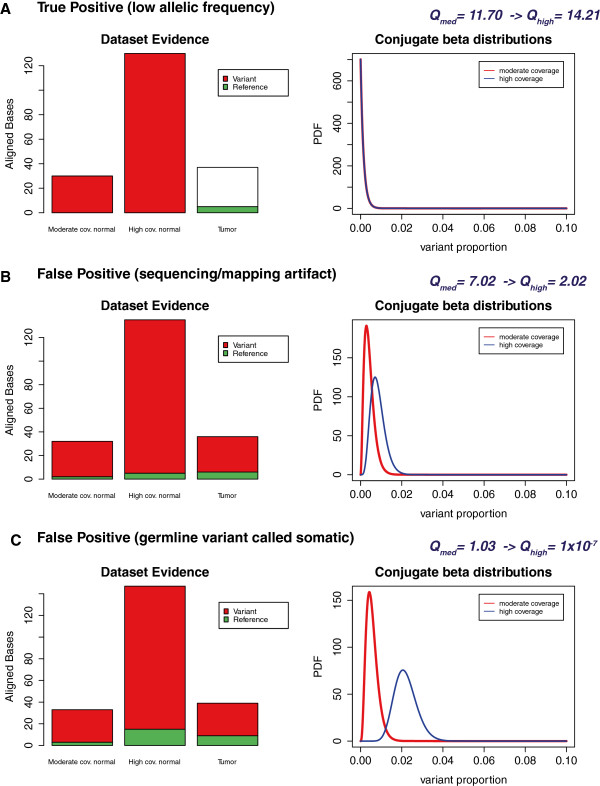
**The effect of increased sequencing of the normal genome on Seurat’s somatic mutation detection.** Legend: Demonstration of the effect of increased sequencing of the normal genome in a matched normal/tumor analysis using Seurat. We present three common scenarios: **A)** a locus with a true somatic variant, but presented with low frequency of the variant allele, because of mapping difficulty, low purity of the tumor biopsy or because of the variant being present only in a minor sub-clonal population. **B)** a locus with a potential false-positive call, because of erroneously-aligned variant evidence. **C)** a locus with a variant genotype in the normal genome, but with a coincidental lack of evidence causing it to appear as a tumor-only variant. In all three scenarios, the increase in sequencing data available for the normal genome updates the expectation of variant evidence (by altering the shape of the conjugate beta distribution) and consequently amplifies Seurat’s capability to correctly reject the last two cases and accept the first case.

Seurat uses the data in the normal genome to ‘update’ the beta-distribution used for the detection of somatic mutations, meaning that an increase in sequencing coverage in either normal or tumor genomes will benefit accuracy. Figure 3 demonstrates how additional sequencing of the normal genome can help with both rejection of false positives and the detection of true positives of low allelic frequency. Though this can be a useful attribute when only the normal tissue is available for additional sequencing runs, sensitivity will still remain low if the tumor alignments do not yield more than 2–3 reads containing the somatic variant (if, by chance, the mutated allele is not sampled enough). When deciding on the amount of sequence to produce, it is therefore useful to consider (and if possible, to estimate) the purity of the tumor tissue or the possibility of tumor heterogeneity. With normal-tissue contamination at 50%, there will be an expected ~6% decrease in sensitivity if the coverage averages 128×; the drop will be higher for a lower average coverage.

We have demonstrated how our methods are able to accurately detect a variety of somatic events that are linked to cancer such as point mutations (base substitutions, insertions and deletions), LOH events between genomes and certain types of structural variation such as translocations and large deletions.

The Bayesian framework used in Seurat is not limited to analysis of DNA mutations. It’s also possible to use the tumor’s sequenced transcriptome to detect allelic imbalance events in a set of known and annotated transcripts by evaluating the probability of a significant shift in the distribution of heterozygous bases. If the aligned transcriptome of the normal tissue is provided, one will be able to detect allelic imbalance events that only occur in the tumor. Otherwise, the tumor DNA will be used to establish normal distribution of heterozygous evidence instead, and the results may include unbalanced alleles that also exist in non-cancerous cells. These approaches represent currently available experimental analysis within Seurat, and are mentioned to demonstrate the generalizable nature of the Bayesian-based analysis scheme. The immediate challenge with determining such priors is that lack of experimental systems with known truth allele specific shifts. Future efforts will be needed to optimize appropriate priors for detection of allele specific expression that specifically capture the unique characteristics of comparing RNA and DNA derived data aligned separately through independent pipelines.

## Conclusions

In summary, paired-genome sequencing in cancer can present us with a highly accurate view of how the cancer genome has evolved from a normal cell’s DNA. It is then beneficial to cancer genomic research that we continue the exploration of paired-genome analysis algorithms, in order to extract a clearer picture of a tumor’s profile and even its evolutionary narrative.

## Methods

### Evidence classes

For the purposes of our methods, evidence is grouped by the genotype that they indicate. Non-variant alignments are ones that do not point to a change from the reference genome while variant alignments are split into subclasses. Each subclass stands for a specific genotype change that is being proposed, and each subclass is then sequentially tested. Classifying all evidence in a binary fashion (“supporting variant” versus “supporting non-variant”) allows us to regard each piece of evidence as a Bernoulli trial, where a success is evidence for a specific change, and failures are everything else.

### Simulated data

In order to test accuracy of our methods, we emulated the existence of somatic point mutations by comparing two unrelated human genomes. We used publically-available exome sequence data from the 1000 Genomes project for this purpose (available from http://www.1000genomes.org). We chose the samples NA19240 and NA12878 as “normal” and “tumor”, respectively. The sequence data was generated using Illumina instruments, and aligned using the MOSAIK software package. For our variant truth set, we used validated genotype calls that are available for these same individuals from the Hapmap project (International HapMap Consortium 2003). From these genotype lists we extracted a list of true positives (sites where the normal genotype matches the reference, but the tumor genotype does not), and two lists of true negatives (sites where both samples match the reference, and sites where both samples do not).

To simulate normal-tissue contamination and generally low allelic frequencies in the presentation of somatic variants, we developed an in-house tool based on the Picard SAM manipulation library (http://picard.sourceforge.net) that randomly selects aligned reads from the two alignment data files at a user-specified ratio and creates a new admixed dataset. We used this to create created new BAM files for a range of simulated tumor purity ratios. The new datasets can then be paired with the individual that was tagged as ‘normal’, and given as input to the software.

### Somatic mutation detection

We define the probability of a somatic mutation event (P(somaticSNV)) as the joint probability of a non-variant genotype being detected in the normal genome (P(ref_normal_)) and a variant genotype being detected in the tumor genome P(¬ref_tumor_).

$$\begin{array}{l} P\left(\mathrm{somaticSNV}\left|D_{\mathrm{normal}}{,D}_{\mathrm{tumor}}\right)\right)=P\left({\mathrm{ref}}_{\mathrm{normal}}\left|D_{\mathrm{normal}}\right)\right)\\ \;\times P\left({\neg \mathrm{ref}}_{\mathrm{tumor}}\left|{\mathrm{ref}}_{\mathrm{normal}}{,D}_{\mathrm{normal}}{,D}_{\mathrm{tumor}}\right)\right) \end{array}$$

In the case of detecting somatic point mutations, each nucleotide of the reference sequence is evaluated independently given that it is sufficiently covered by aligned sequence on both normal and tumor genomes (by default , we take sufficient coverage to be a minimum of 5 aligned bases that pass base and mapping quality filtering). D_normal_ and D_tumor_ are the sets of mapped bases (base pileups) for the normal and tumor genome, respectively. Interpreting the base pileups as Bernoulli trials, a success signifies an alignment that differs from the reference sequence (base mismatch, or a read alignment with an insertion/deletion edit at the tested site), and a failure is a mapped base that matches it.

Given that the examined locus is homozygous and matches the reference, the success probability of the Bernoulli trials is expected to be near zero and the genotyping error rate of the sequencing instrument. This success proportion can, however, be highly variable - because of possible systematic sequencing and aligner software biases, as well as variability in the mappability of the reference sequence.

We use a beta-binomial distribution to model the probability of the evidence, given a genotype that matches the reference. The beta-binomial distribution uses a beta distribution as a probability density function for the proportion of success (variant); this allows us to model the uncertainty of its true value.

$$\begin{array}{l} P\left(D_{\mathrm{normal}}\left|{\mathrm{ref}}_{\mathrm{normal}}\right)\right)={\mathrm{beta}\_\mathrm{binomial}}_{\mathrm{pmf}}\\ \;\left(N_{\mathrm{normal}}{,K}_{\mathrm{normal}}{,\alpha }_{\mathrm{ref}}{,\beta }_{\mathrm{ref}}\right) \end{array}$$

The hyperparameters α and β of the beta distribution in this case are set so as to skew the curve to zero. These parameters can be adjusted at the command line using any additional knowledge of the error profile.

We then apply Bayes’ theorem to extract a probability for homozygosity. Since we can assume that the normal genome is diploid, the marginal probability of the evidence (P(D_normal_)) can be taken to be the sum of the likelihoods of the evidence given the three possible genotype classes (homozygous matching the reference, homozygous variant to the reference, and heterozygous). The selected default values for the prior probabilities for each genotype (πi), as well as the hyperparameters α_ι_ and β_ι_ are listed in Table 3.

$${P(\mathrm{ref}}_{\mathrm{normal}}\left|D_{\mathrm{normal}})=\frac{\pi_{\mathrm{ref}}\times P\left(D_{\mathrm{normal}}\left|{\mathrm{ref}}_{\mathrm{normal}}\right)\right)}{\sum_{i}^{G}\mathrm{\pi i}\times P\left(D_{\mathrm{normal}}\left|i\right)\right)}\right)$$

**Table 3:** Description of priors used in Seurat

**Symbol**	**Description**	**Default values**
π_ι_	Genotype prior probabilities	π_var_ = 0.0005
		π_het_ = 0.001
		π_ref_ = 1 – (π_het_ + π_var_) = 0.9985
		π_somatic =_ 0.0001
		π_LOH =_ 0.0001
α_ι_, β_ι_	Alpha and beta hyperparameters for the beta distributions of variant allele proportions	α_ref_ = 1, β_ref_ = 700
		α_var_ = 700, β_var_ = 1
		α_nonhom_ = 1, β_nonhom_ = 1
		α_somatic_ = 1, β_somatic_ = 1
		α_AI_ = 1, β_AI_ = 1

Detailed Legend: A list of the priors and hyperparameters used by Seurat, and their assigned values. The priors used for the genotype in the normal genome are the SNP frequencies for human diploid chromosomes, as calculated by Li et al. [24]. π_somatic_ and π_LOH_ are high-end estimates of the frequency of somatic events, given that that the mutation profile of each individual cancer can vary wildly even within subtypes. At 0.0001, they expect 300,000 events through the human genome.

The second half of the method is a similar calculation with two major differences. Firstly, we are now looking at the cancer genome so we will no longer assume diploidy or an expected allele frequency. Therefore we use the uninformative beta distribution with parameters [α = 1, β = 1] for variants, and the genotype classes are reduced to just “reference-homozygous” and “somatic variant”.

$$\begin{array}{l} P\left(D_{\mathrm{tumor}}\left|{\neg \mathrm{ref}}_{\mathrm{tumor}}{,\mathrm{ref}}_{\mathrm{normal}}\right)\right)\\ \;=\mathrm{beta}\_{\mathrm{binomial}}_{\mathrm{pmf}}\left(N_{\mathrm{tumor}}{,K}_{\mathrm{tumor}}{,\alpha }_{\mathrm{somatic}}{,\beta }_{\mathrm{somatic}}\right) \end{array}$$

Secondly, we can use the evidence observations from the normal genome to ‘update’ our beta distribution used for the reference homozygosity calculation. This is a simple case of adding the count of successes and failures to the α and β parameters respectively.

$$\begin{array}{l} {P(D}_{\mathrm{tumor}}|{\mathrm{ref}}_{\mathrm{normal}}{,\mathrm{ref}}_{\mathrm{tumor}})=\mathrm{beta}\_{\mathrm{binomial}}_{\mathrm{pmf}}\\ \;\left(N_{\mathrm{tumor}}{,K}_{\mathrm{tumor}}{,\alpha }_{\mathrm{ref}}{+K}_{\mathrm{normal}}{,\beta }_{\mathrm{ref}}{+N}_{\mathrm{normal}}{-K}_{\mathrm{normal}}\right) \end{array}$$

This property of beta distributions helps to overcome the imprecision of priors and the variability of the error rate; in the case of very high-coverage data such a targeted sequencing or whole-exome projects, the normal evidence can virtually overcome the prior beta distribution and allow for sensitive and specific detection of very low-frequency variants. Finally, Bayes’ Theorem yields:

$$\begin{array}{l} P\left({\neg \mathrm{ref}}_{\mathrm{tumor}}|{\mathrm{ref}}_{\mathrm{normal}}{,D}_{\mathrm{normal}}{,D}_{\mathrm{tumor}}\right)\\ \;=\frac{\pi_{\mathrm{somatic}}\times P\left(D_{\mathrm{tumor}}|{\neg \mathrm{ref}}_{\mathrm{tumor}}{,\mathrm{ref}}_{\mathrm{normal}}\right)}{\sum_{i}^{G}\pi_{i}\times P\left(D_{\mathrm{tumor}}|{i,\mathrm{ref}}_{\mathrm{normal}}\right)} \end{array}$$

### Detection of somatic loss of heterozygosity

Testing for somatic loss heterozygosity in our methodology would come from the joint probability of a non-homozygous genotype in the normal genome and a homozygous genotype in the tumor genome (either reference-homozygous or variant-homozygous). The presentation of a non-homozygous genotype in the tumor genotype is once again updated using the evidence from the normal:

$$\begin{array}{l} P\left(\mathrm{somaticLOH}\left|D_{\mathrm{normal}},D_{\mathrm{tumor}}\right)\right)\\ \;=P\left(\mathrm{he}t_{\mathrm{normal}}\left|D_{\mathrm{normal}}\right)\right)\\ \;\times P\left(\mathrm{\neg he}t_{\mathrm{tumor}}\left|\mathrm{he}t_{\mathrm{normal}},D_{\mathrm{tumor}}\right)\right) \end{array}$$

$$\begin{array}{l} P\left({\neg \mathrm{het}}_{\mathrm{tumor}}|\mathrm{he}t_{\mathrm{normal}},D_{\mathrm{normal}},D_{\mathrm{tumor}}\right)\\ \;=\frac{\mathrm{\pi LOH}\times P\left(D_{\mathrm{tumor}}|{\neg \mathrm{het}}_{\mathrm{tumor}},{\mathrm{het}}_{\mathrm{normal}}\right)}{\sum_{i}^{G}\pi_{i}\times P\left(D_{\mathrm{tumor}}|{i,\mathrm{het}}_{\mathrm{normal}}\right)} \end{array}$$

The presentation of a non-homozygous genotype in the tumor genotype is once again updated using the evidence from the normal:

$$\begin{array}{l} P\left(D_{\mathrm{tumor}}\left|{\mathrm{het}}_{\mathrm{normal}},\mathrm{he}t_{\mathrm{tumor}}\right)\right)\\ \;={\mathrm{beta}\_\mathrm{binomial}}_{\mathrm{pmf}}\left(N_{\mathrm{tumor}}{,K}_{\mathrm{tumor}},\alpha_{\mathrm{ref}}\right)\\ \;\left({+K}_{\mathrm{normal}}{,N}_{\mathrm{normal}}{-K}_{\mathrm{normal}}\right) \end{array}$$

### Allelic imbalance detection

We use a similar method to somatic point mutation discovery in order to compute the probability of allelic expression imbalance of a transcript (AI). Starting with paired normal and tumor RNA alignment data, the mapped bases for each nucleotide in the reference sequence are once again translated to Bernoulli successes and failures. A Bayes factor is then used to compare the likelihoods of the tumor transcript data a) if the variant allele proportion is equal to the proportion of the normal sample at the same locus (no somatic AI) and b) if the proportions are independent.

$$P\left(D_{\mathrm{tumor}}\left|M_{2},G,D_{\mathrm{normal}}\right)\right)=\left\{\begin{array}{l} \mathrm{beta}\_\mathrm{binomia}l_{\mathrm{pmf}}\left(K,N,\alpha_{G},\beta_{G}\right),\;G\to \left\{\mathrm{Ref},\mathrm{Var}\right\}\\ \;\mathrm{beta}\_\mathrm{binomia}l_{\mathrm{pmf}}\left(K,N,\alpha_{\mathrm{AI}},\beta_{\mathrm{AI}}\right),\;G\to \left\{\mathrm{Het}\right\} \end{array}\right)$$

$$\begin{array}{l} P\left(D_{\mathrm{tumor}}\left|M_{1},G,D_{\mathrm{normal}}\right)\right)\\ =\left\{\begin{array}{l} \mathrm{beta}\_\mathrm{binomia}l_{\mathrm{pmf}}\left(K,N,\alpha_{G},\beta_{G}\right),\\ \;G\to \left\{\mathrm{Ref},\mathrm{Var}\right\}\\ \;\mathrm{beta}\_\mathrm{binomia}l_{\mathrm{pmf}}\left(K,N,K_{\mathrm{normal}},N_{\mathrm{normal}}-K_{\mathrm{normal}}\right),\\ \;G\to \left\{\mathrm{Het}\right\} \end{array}\;\right\} \end{array}$$

$$K=\frac{\sum_{i}^{G}\mathrm{\pi i}\times P\left(D_{\mathrm{tumor}}\left|M_{1},i,D_{\mathrm{normal}}\right)\right)}{\sum_{i}^{G}\mathrm{\pi i}\times P\left(D_{\mathrm{tumor}}\left|M_{2},i,D_{\mathrm{normal}}\right)\right)}$$

The implementation requires that gene annotations are provided, which are used to limit the process only to loci where reads have aligned within a known transcript. For each sufficiently covered nucleotide in a transcript region the likelihood calculations are performed, and the prior odds are multiplied with the Bayes factor K to give us the updated (posterior) odds.

## Abbreviations

LOH: Loss of heterozygosity; NGS: Next-generation sequencing; indel: Insertion/deletion; GATK: Genome Analysis Toolkit; FDR: False discovery rate.

## Competing interests

The authors declare that they have no competing interests.

## Authors’ contributions

AC co-designed and implemented the somatic mutation detection methods, and carried out the analyses described in the paper. JDC provided feedback, guidance and testing of the algorithms and their implementation. GJW and MJD provided the experimental dataset and participated in study design, whose analysis results were reported on in this paper. DWC co-designed the somatic mutation detection algorithms and provided feedback and guidance throughout development. All authors read and approved the final manuscript.

## Supplementary Material

Additional file 1 Supplementary information. Click here for file
