# Supplementary material for: Identification of somatic mutations in cancer through Bayesian-based analysis of sequenced genome pairs
Source: BMC Genomics. 2013 May 4;14:302. doi: 10.1186/1471-2164-14-302 (PMC3751438; doi:10.1186/1471-2164-14-302)
Supplement: Additional file 1 — Supplementary information. [file 1471-2164-14-302-S1.pdf]

Supplementary Information

Execution/configuration information for the software compared in the section "Effect of heterogeneity on detection performance"

Effort has been made to use each program following its specification and documentation. The command line/configuration used is described below, as well as justification for any changes.

For the command lines that follow, *$1* is a stand-in for the normal BAM file, *$2* for the tumor BAM file, and *$3* for the output file or directory.

Seurat v. 2.2

```
java -jar ./bin/Seurat.jar -T Seurat -R
human_g1k_v37_short_sequence_name.fasta -I:dna_normal $1 -I:dna_tumor $2 -o
$3  -L truth_set/illumina_exome_cov_6/hapmap_autosomal.ALL.interval_list
```

Strelka v. 0.4.3

Note: The configuration file used was the default provided with the Strelka package for the BWA aligner (strelka_config_bwa_default.ini). The parameter "isSkipDepthFilters" was set to 1 as per https://sites.google.com/site/strelkasomaticvariantcaller/home/faq

```
strelka_workflow/configureStrelkaWorkflow.pl --normal=$1 --tumor=$2 –
ref=human_g1k_v37_short_sequence_name.fasta --
config=strelka_config_bwa_default.ini --output-dir=$3

cd $3

make -j 8
```

VarScan v. 2.3.4

Note: VarScan results can contain germline/LOH events even in somatic mode. These are manually filtered out and are not counted as false positives.

```
java -jar ./bin/VarScan.v2.3.4.jar somatic  $1.pileup $2.pileup $3 --output-
vcf --min-coverage-normal 6 --normal-purity $4 --tumor-purity $5
```

$4 and $5 are values for expected normal and tumor purity (1.0 being the default for both, meaning zero contamination). Since a) VarScan does not include a method to estimate the tumor purity and b) the other software is agnostic to the true level (and dimensionality) of tumor heterogeneity, these values were left to their defaults.

SomaticSniper v. 1.0.2

```
./bin/bam-somaticsniper -F vcf -f human_g1k_v37_short_sequence_name.fasta $2
$1 $3 -q 10 -Q 10
```

Prior Selection for Seurat

Genotype Priors

The priors used for the genotype in the normal genome are the SNP frequencies for human diploid chromosomes, as calculated by (Li et al. 2009):

$\pi_{het} = 0.001$

$\pi_{var} = 0.0005$

$\pi_{ref} = 1 - (\pi_{het} + \pi_{var}) = 0.9985$

$\pi_{somatic}$ and π_{LOH} are high-end estimates of the frequency of somatic events, given that that the mutation profile of each individual cancer can vary wildly even within subtypes. At 0.0001, they expect 300,000 events through the human genome.

References

Li, Ruiqiang, Yingrui Li, Xiaodong Fang, Huanming Yang, Jian Wang, Karsten Kristiansen, and Jun Wang. 2009. "SNP Detection for Massively Parallel Whole-genome Resequencing." *Genome Research* 19 (6) (June): 1124–1132. doi:10.1101/gr.088013.108.

Seurat Documentation

Usage

Seurat is a command-line Java application, packaged as a stand-alone JAR file. It is compatible with any operating system platform which is support by the Sun Java 1.6 runtime (including Linux, Windows, and Mac OS X).

Seurat can be executed using a command prompt (terminal) window of the operating system, by moving to the directory of the JAR file and executing the following command:

```
java -jar Seurat.jar -T Seurat -R (reference sequence FASTA file) -I:dna_normal (path to the indexed BAM of normal genome) -I:dna_tumor (path to the indexed BAM of tumor genome) -I:rna_normal (path to the indexed BAM of normal RNA BAM)] [-I:rna_tumor (path to the indexed BAM of tumor RNA BAM)] -o somatic_variants.vcf -go large_events.txt [ARGUMENTS...]
```

[ARGUMENTS...] are options picked from the section below.

Supported arguments

Required:

-o <out> Output VCF.

-go <gene_out> Tab-delimited text output for non-focal events. Most large event analyses

require the 'refseq' argument below.

Optional:

--indels Enable somatic insertion/deletion calling. Default = false

-refseq <refseq_file> Name of RefSeq transcript annotation file. If specified,

gene-wide events can be detected, and SNVs/LOH events will be annotated with the

gene name.

-Q <integer> Minimum phred-scale for reported events. Default = 10.

-mbq <integer> Minimum base quality required to consider a base for calling.

Default = 10.

-mmq <integer> Minimum mapping quality for reads to be considered in the pileup.

Default = 10.

-ref <true/false> If true, only reference-matching homozygous positions are allowed on the normal, for SNV discovery. Reduces false positives due to faulty alignments. Default = true.

-alpha <integer> Alpha parameter of the beta-distribution used for evaluating

homozygosity likelihood. Default = 1.

-beta <integer> Beta parameter of the beta-distribution used for evaluating

homozygosity likelihood. Default = 701.

--both_strands Whether or not variant evidence needs to appear on both strands on the tumor in order to be considered. Default = false.

-coding_only Reduces full-genome and transcript analyses to coding regions of genes. Requires the **–refseq** argument. Default = false.

-mm Maximum number of mismatches against the reference that are allowed in a read. Reads surpassing this number are filtered out. Can be used as an attempt to salvage 'dirty' BAMS containing large numbers of problematic and unlikely alignments, usually due to bugs in the aligner software.) Default =3.

-pileup Enable full pileup output for each call in the VCF file. Default = false

-**mcv <integer>** The minimum per-sample coverage required to attempt a call at a locus. Default = 6

Usage notes / Known Issues

- We recommend the use of the GATK Indel Realigner to jointly process the normal and tumor DNA BAMs for Seurat, as we have empirically found that it reduces indel false positive counts significantly.

- We have found that the Base Quality recalibrator provides a minimal accuracy improvement.

- We do not recommend the use of the base alignment quality (BAQ) (which can be enabled if needed with the '-baq' argument). BAQ currently appears to be causing a significant drop in sensitivity.

- We do not recommend the use of the Variant call recalibrator on Seurat results, as the tool was not designed for somatic calls.

- Seurat accepts most global GATK arguments that can affect its functions. For more information on the GATK framework, please visit http://www.broadinstitute.org/gsa/wiki/index.php/The_Genome_Analysis_Toolkit.

- Seurat does not support the '-nt' option for running multiple threads within GATK. However, the GATK interval option ("-L") can be used to split the data into "bins" that can run simultaneously.

- If Seurat runs without any errors, but the output files do not contain any calls, please check the following:

a) Read group tags ("@RG") are required for all BAMs that are provided; BAMs without RG tags will be ignored (more accurately, any reads that are not assigned in a read group will not be used for analysis). If your BAMs were not generated with RG tags, you may use the Picard tool AddOrReplaceReadGroups to add them. **Please note (b) below if you have to add read groups manually.**

b) The same sample name ("SM") **cannot** be used on read groups belonging on both the normal and the tumor samples. GATK currently uses sample names to group alignments together, so if they are identical between datasets, they will be merged in-memory.

- BAM files for analysis must match **exactly** on their header's sequence names, sequence order, and sequence length.

- For more information on how GATK handles BAM files, please refer to http://gatkforums.broadinstitute.org/discussion/1317/collected-faqs-about-bam-files

Example / Suggested use

```
java -jar Seurat.jar -T Seurat -R ref.fasta -I:dna_normal DNA_normal.BAM -
I:dna_tumor DNA_tumor.BAM -I:rna_tumor RNA_tumor.BAM -o somatic_variants.vcf
-go large_events.txt -Q 15 -refseq refseq.rod
```

Output formats

VCF (-o):

The text file follows the Variant Call Format (VCF) version 4.1, with one line per call. Please refer to the VCF format definition at http://www.1000genomes.org/wiki/Analysis/Variant%20Call%20Format/vcf-variant-call-format-version-41 for more information.

Output will look like the following records:

```
chr2 51001432 . G C 13.7 PASS TYPE=somatic_SNV;PILEUP=ggggGCGG/ccgGgcCgGcgGccCgg;DP=17
```

```
chr2 204596009 . T G 20.4 PASS
TYPE=somatic_SNV;PILEUP=TttTtttttTttTtttttTTTttTtt/TTttttTtTttttttTTtTTTTTTTTTtGtTTtTtttgTGgGtt;DP=26

chr2    40279008    .   A   <DEL>   11.4    PASS    TYPE=somatic_deletion;PILEUP=aaaaAAAAAaaD/aADddddaaa;DP=10
```

The TYPE tag in the INFO field describes the somatic event that was detected.

The types currently are somatic_SNV, somatic_deletion, somatic_insertion and LOH.

The ALT genotype describes either the variant detected in the tumor genome (in case of a somatic SNV event), or the variant allele that is lost in the tumor (in case of an LOH event). The strings "<INS>" and "<DEL>" represent indels.

Large event list (-go):

A simple two-field tab-delimited text file in the following format:

```
[region]    [gene name] [event name/description]    [quality]   [additional info fields]
```
